# 

**Published:** 1874-04

**Authors:** 


					﻿TERMS, ALWAYS IN ADVANCE: One copy, per annum............................. $300
Yearly subscriptions must begin with the January number.
Persons who send Subscriptions for the Journal will please consider the first number
received after subscription as a sufficient receipt for money sent.
We cannot return rejected manuscripts.
Complete Sets for the years 1872 and 1873, will be furnished for S3.00 each.
				

